# Fundamental movement skill proficiency and objectively measured physical activity in children with bronchiectasis: a cross-sectional study

**DOI:** 10.1186/s12890-021-01637-w

**Published:** 2021-08-17

**Authors:** Barbara Joschtel, Sjaan R. Gomersall, Sean Tweedy, Helen Petsky, Anne B. Chang, Stewart G. Trost

**Affiliations:** 1 School of Human Movement and Nutrition Sciences, The University of Queensland, Brisbane, Australia; 2 School of Health and Rehabilitation Sciences, The University of Queensland, Brisbane, Australia; 3 School of Nursing and Midwifery, Griffith University, Brisbane, Australia; 4 Child Health Division, Menzies School of Health Research, Charles Darwin University, Darwin, Australia; 5 Department of Respiratory and Sleep Medicine, Queensland Children’s Hospital, Brisbane, Australia; 6 School of Exercise and Nutrition Sciences, Queensland University of Technology, Brisbane, Australia; 7 QLD Centre for Children’s Health Research (CCHR), Level 6, 62 Graham Street, South Brisbane, QLD 4101 Australia

**Keywords:** Youth, Motor competence, Gross motor skills, Respiratory disease, Exercise

## Abstract

**Background:**

Bronchiectasis is a major contributor to respiratory morbidity and healthcare utilization in children. Children with bronchiectasis exhibit low levels of physical activity (PA) and poor fundamental movement skills (FMS) may be a contributing factor. However, there are no data on FMS’s in this population. The current study assessed FMS proficiency in children with bronchiectasis and examined associations with objectively measured PA.

**Methods:**

Forty-six children with bronchiectasis (mean age 7.5 ± 2.6 year, 63% Male) were recruited from the Queensland Children’s Hospital, Brisbane. PA was measured using the ActiGraph GT3X + accelerometer. Raw accelerometer data were processed into daily time spent in sedentary activities, light-intensity activities and games, walking, running, and moderate-to-vigorous activities and games using a random forest (RF) PA classification algorithm specifically developed for children. Daily MVPA was calculated by summing time spent in walking, running, and moderate-to-vigorous activities and games. FMS were assessed using the Test of Gross Motor Development 2nd Edition (TGMD-2).

**Results:**

Fewer than 5% of children demonstrated mastery in the run, gallop, hop, and leap; while fewer than 10% demonstrated mastery for the two-handed strike, overarm throw, and underarm throw. Only eight of the 46 children (17.4%) achieved their age equivalency for locomotor skills, while just four (8.7%) achieved their age equivalency for object control skills. One-way ANCOVA revealed that children achieving their age equivalency for FMS had significantly higher levels of MVPA than children not achieving their age equivalency (51.7 vs 36.7 min/day). When examined by the five activity classes predicted by the RF algorithm, children achieving their age equivalency exhibited significantly greater participation moderate-to-vigorous intensity activities and games (22.1 vs 10.7 min/day). No significant differences were observed for sedentary activities, light-intensity activities and games, walking, and running.

**Conclusion:**

Children with bronchiectasis exhibit significant delays in their FMS development. However, those who meet their age equivalency for FMS proficiency participate in significantly more daily MVPA than children who do not meet their age-equivalency. Therapeutic exercise programs designed to improve FMS proficiency are thus likely to be beneficial in this population.

## Introduction

Bronchiectasis is a major contributor to respiratory morbidity in children and was recently identified as one of the most neglected lung diseases [1]. It is the end point of the chronic suppurative lung disease continuum and is described as abnormal irreversible dilatation of bronchi and bronchioles, caused by recurring airway infection and inflammation [1, 2]. The occurrence of exacerbations, defined as increased wetness and severity of cough, breathlessness, chest pain, and wheeze lead to frequent hospitalization and further decline in lung function [3]. Prevalence data in children are scarce; however, it is estimated that the prevalence of bronchiectasis ranges from 0.2 cases to 15 cases per 100,000 [4]. Of note, the prevalence of bronchiectasis is higher among socially disadvantaged populations, such as the Indigenous communities of Australia, New Zealand, Alaska and Canada [2, 5].

Current recommendations for the treatment and management of bronchiectasis in children and adults emphasize the importance of regular physical activity (PA) to improve cardiovascular fitness and quality of life (QoL) [6]. However, most children with bronchiectasis do not meet public health recommendations for PA which call for 60 min or more of moderate-to-vigorous PA (MVPA) daily [7]. Using an accelerometer to quantify habitual PA, we observed consistently low levels of daily MVPA in 36 children with bronchiectasis. Only two children (5.6%) achieved the recommended 60 min of daily MVPA. In contrast, 42% of healthy children in the normative comparison group achieved the guideline. Expressed as a percentage of the waking hours, children with bronchiectasis were sedentary for 57.5% of the time, in light-intensity PA 35.8% of the time, and in MVPA just 6.7% of the time [7].

Fundamental movement skill proficiency is an important determinant of children’s current and future PA status, and a significant contributor to individual health and well-being [8, 9]. The development of fundamental movement skills (FMS) early in life is critical to establishing the more complex movement patterns required for participation in all types of games, physical activities, and sports [8]. Children who are proficient in FMS are more likely to participate in and enjoy PA [10], achieve higher levels of cardiovascular fitness [11], exhibit higher levels of perceived competence [12, 13] and self-esteem [9, 14], and are less likely to be overweight or obese [15]. Notably, a 6-year longitudinal study found FMS proficiency during early childhood to be a significant positive predictor of PA participation and cardiovascular fitness during late adolescence [16].

In light of the evidence linking FMS proficiency to current and future PA participation, it seems reasonable to hypothesize that the low levels of PA observed in children with bronchiectasis may be attributable, at least in part, to developmental delays in FMS proficiency. If it can be shown that delays in FMS contribute to the low PA levels of children with bronchiectasis, then therapeutic exercise programs designed to improve FMS proficiency and fitness are likely to be highly beneficial in this population. Current practice standards for pediatric therapy of children with bronchiectasis focus on airway clearance techniques and do not address issues related to movement competence. To our knowledge, the relationship between FMS proficiency and habitual PA in children with bronchiectasis has not been systematically investigated. Therefore, the aims of the current study were to: 1) assess the level of FMS proficiency in children with bronchiectasis; and 2) determine if FMS proficiency is associated with objectively measured PA.

## Methods

### Participant recruitment

Children with bronchiectasis between the ages of 4–13 years were recruited between March 2015 and February 2017 through the Respiratory and Sleep Department at the Queensland Children’s Hospital, Australia. Bronchiectasis was diagnosed according to guidelines published by the Thoracic Society of Australia and New Zealand [5]. Children with unstable medical conditions, unstable emotional or behavioral status, or recent musculoskeletal injuries (e.g., sprain, fracture, muscle strain) were excluded. Clinicians within the department were provided with a detailed description of the study, along with inclusion and exclusion criteria. Parents of potential participants were contacted by the primary investigator who formally assessed eligibility and provided detailed information about the study. After discussing the study, parents with children who were eligible and interested in participating provided written informed consent. In addition to parental consent, children aged between 7 and 13 years provided written informed assent. Ethical approval for this study was received from the Human Research Ethics committee at the Children’s Health Queensland Hospital and Health Service (HREC/14/QRCH/136) and the Human Research Ethics committee at The University of Queensland (2014001176). All research was conducted in accordance with relevant guidelines and regulations.

### Measures

#### Parent questionnaire

Parents provided information about the following: duration of the cough, if the cough was wet or dry, current medications, frequency of doctor visits during the last 12 months, frequency and length of exacerbations, family structure, parental age, parental smoking status and average household income.

#### Fundamental movement skills

FMS were assessed using the Test of Gross Motor Development 2^nd^ Edition (TGMD-2) which measures 12 movement skills in two dimensions: locomotor skills and object control [17]. Scores were based on the achievement of performance criteria for six locomotor (run, gallop, hop, leap, horizontal jump, and slide) and six object control skills (striking a stationary ball, stationary dribble, catch, kick, throw, and roll). All assessments were performed following the standardized protocols in the TGMD-2 examiners manual [17]. For each skill, the performance criteria were rated as “1” (present) or “0” (absent). Ratings were then summed to derive a raw score for the locomotor skills and object control skills, respectively. Mastery status was assigned if all of the observed performance criteria for a given skill were present. If all but one of the criteria were deemed present, performance was defined as near-mastery. The gross motor quotient (GMQ) was derived by summing the locomotor and object control raw scores and converting into a GMQ using the TGMD-2 normative database. The GMQ was then categorized as follows: > 130 very superior, 121–130 superior, 111–120 above average, 90–110 average, 80–89 below average, 70–79 poor and < 70 very poor.

Age equivalents represent the average raw score for individuals between the ages of 3 and 11 years in increments of 3 months. If the child’s raw score was equal to or greater than the published norm (average) for their chronological age interval, they were classified as achieving their age equivalency. If the child’s raw score was less than the published norm for their chronological age interval, they were classified as failing to achieve their age equivalency (development delay). Participants aged 12 or 13 years of age were compared to the published norms for 11-year-olds. The TGMD-2 is a reliable measure of FMS proficiency in children with test–retest reliability ranging from 0.86 to 0.96 [17].

#### Physical activity

PA was measured using the ActiGraph GT3X + accelerometer (ActiGraph Corporation, Pensacola, FL, USA). The accelerometer was worn on the right hip for seven consecutive days during waking hours, except for bathing or water-based activities. Participants were provided with a monitoring log and asked to record sleep, wake, and non-wear times. Monitors were initialized and downloaded using the ActiLife software (Version 6.13.4). Raw accelerometer data (sampling frequency = 30 Hz) were downloaded and processed into physical activity variables using a random forest (RF) physical activity classification algorithm specifically developed for children [18]. The validated RF algorithm uses features extracted from the raw tri-axial acceleration signal (15 s windows or epochs) to quantify daily time spent in sedentary activities (sitting or lying down) (SED), light-intensity activities and games (slow walking/pottering about, standing, standing arts and crafts) (L_ACT_G), walking, running, and moderate-to-vigorous intensity activities and games (active games with balls, riding bikes/scooters) (MV_ACT_G). When applied to new data, recognition accuracy was 98.1% for SED, 95.2% for L_ACT_G, 92.7% for (MV_ACT_G_, 94.5% for walking, and 99.5% for running. Overall classification accuracy was 95.7% [18]. Daily MVPA was calculated by summing daily time spent in walking, running, and MV_ACT_G. Non-wear periods were identified by summing the 15 s windows in which the standard deviation of the acceleration signal vector magnitude was < 13 mg for >  = 30 consecutive minutes [19]. The child’s accelerometer data was included in the analyses if they had ≥ 3 days in which wear time was 8 h or longer.

### Statistical analysis

Descriptive statistics, including means, standard deviations and frequencies were calculated for participant characteristics and the study variables. Differences in average daily MVPA, SED, L_ACT_G, walking, running, and MV_ACT_G between children achieving and not achieving their age equivalency for FMS were evaluated for statistical significance using a one-way ANCOVA, with child sex and accelerometer wear time serving as covariates. Significance was set at an alpha level of 0.05. All statistical analyses were completed using SAS software version 9.4 (SAS Institute Inc., Cary, NC, USA.).

## Results

Of the 55 children with bronchiectasis referred to the study, 46 (83.6%) agreed to participate and were enrolled in the study. Descriptive statistics for the participants are presented in Table 1.

**Table 1:** Descriptive statistics of children with bronchiectasis (n = 46)^a^

	n = 46
Age (years)	7.5 ± 2.6
Male	29 (63.0)
Currently on medication	28 (60.9)
Inhaled steroids	6 (13.0)
Bronchodilators	7 (15.2)
Oral steroids	1 (2.2)
Antibiotics	12 (26.1)
Other	2 (4.3)
*Number of doctor visits in the last 12 months*	
< 5 times	12 (26.1)
5–9 times	18 (39.1)
10–20 times	11 (23.9)
> 20 times	5 (10.9)
Children with a single parent	8 (17.4)
*Average household income*	
< $25,000 AUD	9 (18.2)
$26,000–$50,000 AUD	5 (10.9)
$51,000–$75,000 AUD	1 (2.2)
> $76,000 AUD	31 (67.4)
Families with a smoker	10 (21.7)

^a^Data are expressed as mean ± SD or n (%) of participants

Children were aged (mean ± SD) 7.5 ± 2.6 years, and the majority reported current medication use (60.9%) and > 5 doctor visits during the previous 12 months (73.9%). Overall, 17.4% of the families were single-parent households. Approximately 30% of participating families reported a household income below the Australian poverty line (single adult income of < $426.30/week) [20].

The percentage of children demonstrating mastery, near-mastery and non-mastery for each FMS is reported in Table 2. For the six locomotor skills, mastery ranged from 0.0% to 67.4%, with less than 5% of children achieving mastery in the run (4.3%), gallop (2.2%), hop (0%), and leap (4.3%). For object control, mastery ranged from 4.3% to 30.4%, with less than 10% of the children achieving mastery for striking a ball (8.7%), overarm throwing (4.3%) and underarm rolling (8.7%). The percentage of children accomplishing near-mastery for locomotor and object control skills ranged from 13.0% to 52.2% and 10.9% to 32.6%, respectively. More than two-thirds of the participants exhibited non-mastery status for the hop, strike a ball, dribble, overarm throw, and underarm throw skills. The mean GMQ of 82.7 ± 12.8 indicated that, as a group, FMS performance was well below average.

**Table 2:** Proportion of children with bronchiectasis (n = 46) mastering, nearly mastering or not-mastering fundamental movement skills^a^

	Mastery	Near-Mastery	Non-Mastery
*Locomotion*			
Run	2 (4.3)	22 (47.8)	22 (47.8)
Gallop	1 (2.2)	22 (47.8)	23 (50)
Hop	0 (0.0)	6 (13.0)	40 (87.0)
Leap	2 (4.3)	24 (52.2)	20 (43.5)
Jump	8 (17.4)	17 (37.0)	21 (45.7)
Side slide	31 (67.4)	11 (23.9)	4 (8.7)
*Object control*			
Strike a ball	4 (8.7)	11 (23.9)	31 (67.4)
Dribble	7 (15.2)	17 (17.4)	31 (67.4)
Catch	8 (17.4)	14 (30.4)	24 (52.2)
Kick	14 (30.4)	15 (32.6)	17 (37.0)
Overarm throw	2 (4.3)	5 (10.9)	39 (84.8)
Underarm Roll	4 (8.7)	6 (13.0)	36 (78.3)

^a^Data are expressed as n (%) of participants

Overall, 37 of the 46 children (80.4%) failed to achieve their age equivalency for either locomotor or object control skills. Only eight of the 46 children (17.4%) achieved their age equivalency for locomotor skills, while just four (8.7%) achieved their age equivalency for object control skills.

Table 3 displays means and 95% confidence intervals for the PA outcomes for children achieving and not achieving their age equivalency for either locomotor or object control skills. Of the 46 children participating in the study, 41 provided ≥ 3 valid monitoring days and were included in the ANCOVA analyses (mean number of valid monitoring days = 5.5 ± 1.7 days; 3 children with 3 valid days (7.3%), 27 children with 4 to 6 valid days (65.9%) and 11 children with 7 or more valid days (26.8%). Of 41 children, 9 met their age equivalency for either locomotor or object controls skills, with the remaining 32 children exhibiting locomotor or object control scores below their age equivalency. Children achieving their age equivalency for FMS exhibited significantly higher levels of MVPA than children not achieving their age equivalency (51.7 vs 36.7 min/day). When examined by the five activity classes predicted by the RF PA classification algorithm, children achieving their age equivalency for FMS exhibited significantly greater participation MV_ACT_G (22.1 vs 10.7 min/day). No significant differences were observed for sedentary activities, L_ACT_G, walking, and running.

**Table 3:** Mean (95% Confidence Interval) of physical activity in children meeting their age equivalency and not meeting their age equivalency

PA variable^a^	Age equivalency for FMS proficiency (mean and 95% CI)^b^		Difference (mean and 95% CI)	*P *value
	Meeting (N = 9)	Not meeting (N = 32)		
MVPA^c^ (min/d)	51.7 (45.9–57.5)	36.6 (24.2–49.1)	15.1 (0.3–29.8)	0.04
SED (min/d)	357.9 (312.0–403.8)	356.9 (335.6–378.1)	– 1.0 (− 55.4–53.3)	0.96
L_ACT_G (min/d)	301.2 (260.4–341.9)	287.2 (268.3–306.0)	14.0 (− 62.3–34.2)	0.56
M_ACT_G (min/d)	22.1 (18.1–26.2)	10.7 (2.0–19.5)	11.4 (1.1–21.7)	0.03
WALK (min/d)	23.1 (14.3–32.0)	24.3 (20.2–28.4)	− 1.2 (− 9.3–11.7)	0.82
RUN (min/d)	2.8 (0.2–5.4)	5.3 (4.1–6.5)	− 2.5 (− 0.6–5.6)	0.11

MVPA = moderate-to-vigorous physical activity; SED = sedentary activities; L_ACT_G = light intensity activities and games; M_ACT_G = moderate-to-vigorous intensity activities and games. WALK = walking; RUN = running

^a^Based on the daily average for valid monitoring days. Mean number of valid monitoring days = 5.5 ± 1.7 days

^b^Means adjusted for gender and accelerometer wear time

^c^MVPA calculated by summing daily time in M_ACT_G, WALK, and RUN as classified by a Random Forest PA Classifier [18]

## Discussion

The current study assessed FMS proficiency in children with bronchiectasis and examined the relationship between FMS proficiency and habitual PA. The results show that children with bronchiectasis experience significant developmental delays in FMS proficiency. Just eight of the 46 children achieved their age equivalency for locomotor skills, while just four children achieved their age equivalency for object control skills. Fewer than 5% of the sample demonstrated mastery in the run, gallop, hop, and leap; while fewer than 10% demonstrated mastery for the two-handed strike, overarm throw, and underarm throw.

A major finding of the current study was that FMS proficiency emerged as a significant influence on PA performance in children with bronchiectasis. Children achieving their age equivalency for either locomotor or object control skills exhibited significantly higher levels of daily MVPA than those with developmental delays in FMS. Notably, the differential in daily MVPA was attributable, in large part, to a twofold difference in daily participation in moderate-to-vigorous intensity activities and games. This was an important finding which added an element of ecological validity to the results, given that participation in in this type of activity (e.g., games and sports) would generally require a prerequisite level of FMS proficiency. Notably, there were no significant differences for time in sedentary activities or PA classes known to be less dependent on FMS proficiency (e.g., L_ACT_G, walking and running).

To date, only two previous studies have evaluated FMS proficiency in children with chronic respiratory conditions [21, 22]. In contrast with the results of the current study, both found no evidence of developmental delays in FMS. Gruber et al. [22] assessed motor performance in preschool-aged children with cystic fibrosis (CF) and found motor quotient scores, based on the average of seven motor tasks, to be within the normal range. Similarly, Bender et al. [21] observed no evidence of motor delays in a sample of 67 children with severe chronic asthma. The discrepancy in findings may be explained, in part, by differences in the operational definition and assessment of motor competence. Notably, both studies used assessment batteries that measured both fine and gross motor skills and evaluated skills more closely related to athletic ability (i.e., strength, speed, agility) than FMS proficiency. The results, however, are consistent with previous investigations evaluating FMS proficiency in other pediatric patient groups such as cancer survivors and children with congenital heart disease. Hartmann et al. [23] evaluated movement competency in 120 pediatric cancer survivors. Twelve months post treatment, two-thirds of children scored below the 50^th^ percentile on the movement ABC. Neuman et al. [24] compared the FMS proficiency of pediatric cancer survivors with a reference group of 300 healthy children. Cancer survivors were significantly less likely to exhibit mastery on seven key FMS (sprint run, vertical jump, side gallop, leap, catch, kick, overarm throw) than healthy children. Box and colleagues [25] evaluated the motor performance of 18 children with congenital heart disease. Compared to healthy controls, gross motor performance was significantly delayed. Finally, Holm et al. [26] observed that nearly half of children with complex heart disease had significant motor delays. Of note, none of these studies concurrently measured PA or examined if differences in PA are explained by delays in FMS. Collectively, these findings suggest that children with chronic health conditions are at increased risk for developmental delays in motor proficiency, suggesting that clinicians may need to assess PA levels and evaluate movement competency as part of routine practice; and when indicated, refer patients to developmentally appropriate therapeutic exercise programs to increase movement competency. Clinicians should also counsel children and their parents about the importance of PA and provide them with information to enable them to engage in regular, safe, and enjoyable PA in settings outside therapy.

The observed age delays in FMS proficiency in children with bronchiectasis may be attributable to a number of factors. FMS are not naturally acquired but need to be taught and practiced (31). Therefore, it is possible that periods of inactivity, precipitated by exacerbations and/or the time constraints imposed by frequent medical appointments and therapy sessions may limit opportunities to practice and refine movement skills. Lack of parental support for PA may be another reason, as overprotective parents may discourage participation in sport and PA programs believing that exercise will provoke coughing and cause physical discomfort [7]. Importantly, the relationship between FMS proficiency and physical activity is bi-directional in nature, particularly among young children. While FMS proficiency is essential for participation in many organized and non-organized games and sports, children also require opportunities to learn and practice FMS through regular engagement in developmentally appropriate physical activity.

The current study has several strengths. To our knowledge, it is the first study to systemically evaluate the relationship between FMS proficiency and PA in children with bronchiectasis. FMS proficiency was measured using the TGMD-2, a widely-used and validated process-oriented assessment tool with published norms for both object control and locomotor movement skills. In addition, PA was measured objectively using a wearable sensor and the PA outcomes were derived from raw acceleration signal using state-of-the-art machine learning data processing methods [27]. The application machine learning methods allows researchers to monitor not only the intensity of physical activity, but also the quality of movement behaviors. In contrast to traditional cut-point methods, which simply estimate time spent in moderate-to-vigorous PA, the RF classifier deployed in the current study allowed monitoring of active game play and sports as a component of overall participation in MVPA. This was key given that participation in active games and sports requires greater FMS proficiency than walking and running.

Opposing these strengths were a number of limitations. First, due to the relatively small sample size, we were not able to conduct multivariate analysis controlling for the effects of disease severity, medication use, and socioeconomic status. Second, the cross-sectional study design means it is not possible to infer causal relationships between FMS proficiency and PA participation. Third, the RF PA classification model used to measure daily MVPA was trained on laboratory-based activity trials which may not fully replicate PA performance under true free living conditions [28]. Fourth, age equivalency was based on normative data from North America rather than Australia. In the absence of norms based on a large and representative sample of Australian children, the TGMD normative database was the best available method for determining age equivalents. Lastly, participants were recruited from a single public hospital in Brisbane, Australia and cannot be considered representative of all children with bronchiectasis. In addition, because 9 of the children referred to the study by their physician opted not to participate, and because children with less than 3 valid monitoring days were excluded from the analysis (n = 5), we cannot rule out the possibility of selection bias (i.e., physically active children more likely to be included in the analytic sample than low-active children). However, given the small number excluded, and the generally low levels of FMS proficiency and PA levels in our sample, this is unlikely. Future studies should evaluate the relationship between FMS proficiency and PA levels in larger, more representative samples of children with bronchiectasis. Samples should be sufficiently large and diverse to determine if the relationship between FMS proficiency and PA is moderated by demographic, socioeconomic status, and health characteristics. Future studies should also consider other important potential physiological determinants of habitual PA, including cardiorespiratory fitness, body composition, functional muscle strength, and fatigue. Considering the challenges associated with recruiting large samples of clinical populations, multi-site trials involving multiple hospitals and health systems may be required.

## Conclusion

Children with bronchiectasis exhibit significant delays in their FMS development. However, the small proportion of children who met their age equivalency for FMS exhibited significantly more daily MVPA than children who did not meet their age-equivalency. These findings are consistent with studies conducted in other patient groups and underscore the need for developmentally appropriate therapeutic exercise programs targeting FMS proficiency and other determinants of physical activity behavior among children with chronic health conditions.

## Data Availability

The datasets used and/or analyzed during the current study are available from the corresponding author on reasonable request.

## Abbreviations

PA: Physical activity
FMS: Fundamental movement skills
GMQ: Gross motor quotient
MVPA: Moderate- to vigorous-intensity physical activity
SED: Sedentary activities
L_ACT_G: Light intensity activities and games
M_ACT_G: Moderate-to-vigorous intensity activities and games.
WALK: Walking
RUN: Running
